# Complete Genome Sequence of *Bacillus subtilis* J-5, a Potential Biocontrol Agent

**DOI:** 10.1128/genomeA.00275-17

**Published:** 2017-06-08

**Authors:** Zhenhua Jia, Weiwei Jin, Yali Huang, Shuishan Song

**Affiliations:** a Institute of Biology, Hebei Academy of Sciences, Shijiazhuang, People’s Republic of China; b Hebei Engineering and Technology Center of Microbiological Control on Main Crop Disease, Shijiazhuang, China; c School of Chemical Engineering and Technology, Hebei University of Technology, Tianjin, People’s Republic of China

## Abstract

*Bacillus subtilis* J-5 was isolated from tomato rhizosphere soil and exhibited strong inhibitory activity against *Botrytis cinerea*. To shed light on the molecular mechanism underlying the biological control on phytopathogens, the whole genome of this strain was sequenced. Genes encoding antimicrobial compounds and the regulatory systems were identified in the genome.

## GENOME ANNOUNCEMENT

*Bacillus subtilis* has been widely described as an important biocontrol strain. It has the ability to suppress phytopathogens by producing antimicrobial compounds, such as antibiotics, lantibiotics, and bacillibactin, competing for nutrients and niches with pathogens, and inducing systemic resistance to pathogens (1–3). In addition, *B. subtilis* is approved to promote plant growth (4). Recently, we isolated a strain from tomato rhizosphere soil. This strain has strong inhibitory effect on the growth of *Botrytis cinerea*, a fungal pathogen causing gray mold in tomato. Biochemical and molecular biological analyses assigned this strain to *B. subtilis* J-5. The lipopeptides were identified from the culture broth of *B. subtilis* J-5. Moreover, the volatile substances of *B. subtilis* J-5 were demonstrated to strongly inhibit the growth of *B. cinerea in vitro*. To gain more knowledge on the genetic equipment of this bacterium and provide more insight into the mechanism by which this bacterium plays its biocontrol roles, we sequenced and annotated the complete genome sequence of this strain.

Whole-genome sequencing of strain J-5 was performed on the PacBio RSII sequencing platform at Novogene (Beijing, China). The genomic DNA was randomly sheared to 10-kb target size using PacBio RSII (5). It gives 87 million bases with approximately 211-fold genome sequence coverage. The reads were assembled with SMRT Portal 2.03 software (6). We used GeneMarkS (7) (http://topaz.gatech.edu/) to predict bacterial coding genes. Genomic islands was predicted using software IslandPath-DIOMB. The rRNAmmer software was used to predict rRNAs, the tRNAscan software was employed to predict tRNA regions and tRNA secondary structure, and the Rfam software was used to predict small RNAs (sRNAs). The secondary metabolite gene cluster was identified using the antiSMASH program (8). Gene annotation was added using the NCBI Prokaryotic Genome Annotation Pipeline (9).

The complete genome sequence of *B. subtilis* J-5 is characterized by a circular chromosome of 4,117,900 bp, with a mean G+C content of 46.11%. The chromosome contains 4,312 coding genes, 87 tRNAs, 27 rRNAs, and 9 sRNAs. No plasmid was found in this strain. Genome analysis revealed that the genome of J-5 contains 9 gene clusters devoted to the synthesis of antimicrobial compounds, including polyketide synthase (PKS) antibiotics (type 2 PKS [T2PKS], TransPKS, type 3 PKS [T3PKS], transAT PKS-nonribosomal peptide synthetase [TATPKS-NRPS]), NRPS antibiotics (NRPS-bacteriocin, NRPS), lantibiotics, bacillibactin, and terpene. The two-component signal transduction systems function in response to prokaryotes under a variety of external conditions. It was uncovered that a total of 48 two-component systems (TCS) exist in the genome of strain J-5, including ComP/ComA (10) DegS/DegV (11), QseC/QseB (12), PhoR/PhoP (13), and DesK/DesR (14). The genes encoding RpoS and RpoN (15), which play roles in bacterial adaptation to environmental stress, were also identified in the genome of this strain.

### Accession number(s).

The complete genome sequence of *B. subtilis* J-5 has been deposited at GenBank under the accession number CP018295. The version described in this paper is the first version. This strain has been deposited at the China General Microbiological Culture Collection Center (CGMCC no. 11750).
